# The Impact of Orthodontic Adhesive Containing Resveratrol, Silver, and Zinc Oxide Nanoparticles on Shear Bond Strength: An In Vitro Study

**DOI:** 10.7759/cureus.68346

**Published:** 2024-08-31

**Authors:** Tanya Prasad, Renuka Pawar, Chanamallappa Ganiger, Yusuf Ronad, Sandesh Phaphe, Pratap Mane, Seema Patil

**Affiliations:** 1 Orthodontics and Dentofacial Orthopedics, School of Dental Sciences, Krishna Vishwa Vidyapeeth (Deemed to be University), Karad, IND

**Keywords:** anti-microbial activity, resveratrol nanoparticle, orthodontics, zinc oxide nanoparticle, silver nanoparticles, dentistry, shear bond strength, dentin bonding agent, resveratrol, nanoparticles

## Abstract

Introduction

The goal of orthodontic treatment is to provide patients with esthetic smiles and functional occlusion. Despite best efforts and continuous evolution of materials, white spot lesions present a persistent hindrance to the desired treatment outcome. Nanoparticles have shown efficacy in reducing microbial activity; however, currently, there is a need for natural anti-cariogenic compounds with minimal side effects. Resveratrol is a natural compound belonging to the polyphenol group and has shown promising anti-microbial efficacy. This study aimed to evaluate the influence of dentin bonding agents incorporated with the following three different nanoparticles on shear bond strength: silver nanoparticles (Ag-Np), zinc oxide nanoparticles (ZnO-Np), and resveratrol nanoparticles (RSV-Np).

Materials and methods

A total of 40 premolar teeth therapeutically extracted were assigned to four equal groups of n=10 each. Groups 1, 2, and 3 used experimental adhesives doped with silver, zinc oxide, and resveratrol nanoparticles, respectively. Group 4 was bonded using unmodified adhesive. The bonded teeth were then subjected to shear bond strength (SBS) testing which was measured using a Universal Testing Machine (model no. UNITEST-10; Pune, India: ACME Engineers). Statistical analyses were performed using SPSS version 21 (Armonk, NY: IBM Corp.), employing one-way ANOVA and Tukey’s post-hoc test for pairwise comparisons.

Results

Shear bond strength testing revealed that the control group with unmodified adhesive (8.6 MPa) had the highest SBS, followed by RSV-Np (7.6 MPa), Ag-Np (6.3 MPa), and ZnO-Np (5.65 MPa). Although the experimental groups demonstrated decreased SBS compared to the control, the values for Ag-Np and RSV-Np fell within the acceptable range.

Conclusion

Resveratrol nanoparticles had the least impact on shear bond strength among the experimental groups. These findings suggest that the incorporation of resveratrol nanoparticles in dentin bonding agents can provide anti-cariogenic effect without significantly impacting the adhesive's mechanical properties thereby providing a new and promising alternative to synthetic nanoparticles. Further studies are recommended to optimize the balance between anti-microbial efficacy and bond strength in clinical applications.

## Introduction

Esthetic smiles have always been a hallmark of attractiveness and confidence, playing a crucial role in social interactions and self-esteem. Through various orthodontic treatments, including braces and aligners, orthodontists aim to correct malocclusions and misalignments, ultimately granting patients a harmonious and radiant smile.

Traditionally, bonding of fixed orthodontic appliances has been facilitated by orthodontic adhesive systems, comprising an enamel conditioner, a primer solution, and adhesive resin designed to ensure adherence of the bracket base to the enamel surface.

The conventional bonding to enamel employs the acid etching technique, involving the pretreatment of enamel with 37% phosphoric acid to achieve micromechanical retention on the enamel surface [1]. Subsequently, a primer, dentin bonding agent, and dental adhesive are applied in sequence. As dental materials continue to evolve to enhance clinical practice, the traditional three-step etch-and-rinse adhesive has transitioned into a simplified two-step adhesive, amalgamating the primer and adhesive resin into a single solution. Presently, clinicians favor the etch-and-rinse approach employing 37% phosphoric acid for enamel etching, ensuring both a durable bond to enamel and protection against degradation [2].

Despite advancements in orthodontic adhesive systems, the occurrence of white spot lesions (WSLs) on dental enamel during orthodontic treatment remains a significant challenge in clinical care. Demineralization, followed by white spot lesions formation and the emergence of new caries-prone sites adjacent to bands and brackets, is primarily attributed to low oral pH and lactic acid produced by *Streptococcus mutans* metabolism [3]. Previous studies have documented increased bacterial growth at the interface between adhesive resins used in bonding orthodontic attachments to enamel [4,5]. Moreover, mechanical plaque removal around orthodontic brackets poses a challenge, even with patient compliance.

Various methods have been employed to mitigate enamel demineralization in patients undergoing fixed orthodontic treatment. These include bonding agents with antibacterial properties, mouth rinses containing antimicrobial agents, coatings on brackets/wires, or remineralizing agents adjacent to orthodontic appliances. However, their effectiveness has been observed to be limited [6]. In dentistry, nanotechnology has been harnessed to develop materials with enhanced mechanical properties and anti-bacterial effects [7]. With the advancement of nanotechnology and the unique attributes exhibited by nanoparticles, efforts have been made to use this approach in orthodontic bonding. Incorporating nanoparticles into orthodontic adhesives/cements or acrylic resins and coating them onto orthodontic appliance surfaces has shown promise in preventing microbial adhesion or enamel demineralization in orthodontic therapy [8,9]. The incorporation of nanoparticles into the bonding agent, particularly those in direct contact with the enamel surface, holds particular clinical significance.

Incorporating fluoride, selenium, and various nanoparticles, such as silver, copper, and zinc has been shown to reduce the formation rate of cariogenic biofilm. However, the incorporation of synthetic compounds such as fluoride in toothpastes has been linked to fluorosis due to early exposure or overexposure. This underscores the need for natural anti-cariogenic agents with minimal side effects and optimal effectiveness. Moreover, the rising prevalence of bacteria resistant to commercial anti-microbial agents has underscored the growing demand for natural and non-toxic alternatives [10].

In recent decades, many natural compounds with anti-inflammatory/anti-bacterial properties and minimal toxicity to mammalian tissues have been identified. Among them, trans-resveratrol (RV), a non-flavonoid polyphenol belonging to the class of stilbenes, has emerged as one of the most promising. Recent studies have demonstrated its ability to downregulate inflammatory biomarkers and upregulate anti-inflammatory cytokines [11]. Resveratrol is also known for its antioxidant properties, stimulating cellular antioxidant defense mechanisms and scavenging reactive oxygen species [12]. Moreover, it has been found to be safe and well-tolerated at doses up to 5 g/day, establishing it as a natural compound with potent anti-microbial properties and favorable biocompatibility [13].

Nanotechnology has emerged as the gold standard innovation in dental materials, enhancing their properties and anti-caries potential. Nanomaterials not only combat caries-related bacteria but also effectively reduce biofilm accumulation. Silver nanoparticles exhibit potent anti-microbial properties with low toxicity to human cells. Incorporating silver and zinc oxide nanoparticles into dental adhesive systems enhances their anti-microbial properties while maintaining their mechanical properties [14].

To the best of our knowledge, no previous study has been done to assess the effect of resveratrol on an adhesive's physical properties. Thus, this study aimed to incorporate resveratrol, a natural anti-microbial agent, and evaluate its effect on the shear bond strength of adhesive formulations while comparing it with silver and zinc oxide nanoparticles incorporated adhesives.

## Materials and methods

This in vitro comparative study was conducted at the School of Dental Sciences, Krishna Vishwa Vidyapeeth (Deemed to be University), Karad. This in vitro study protocol received approval from the Institutional Ethics Committee of Krishna Vishwa Vidyapeeth (Deemed to be University), Karad (#KVVDU/IEC/08/2022).

Preparation of experimental adhesive

**Figure 1:**
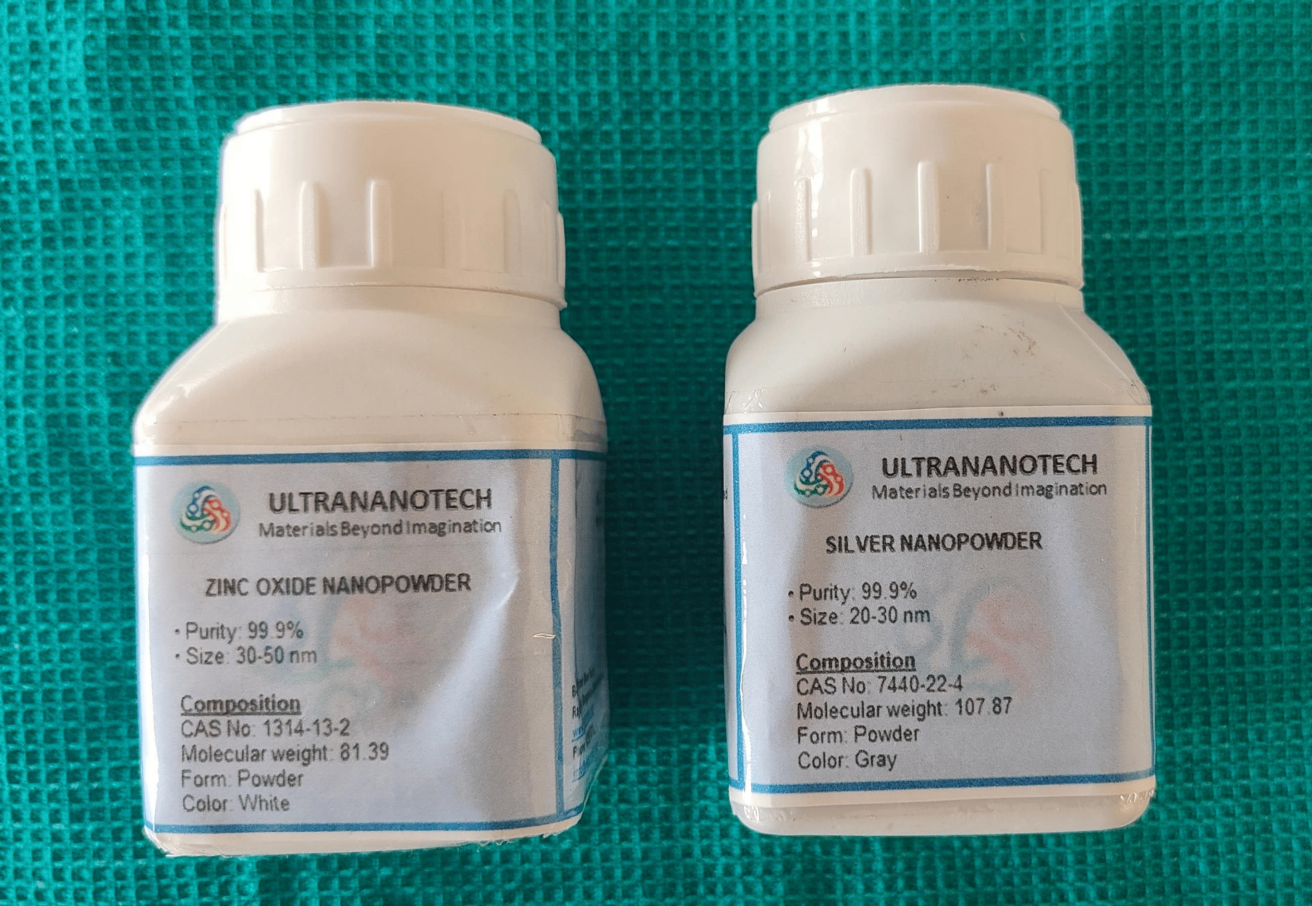
Zinc oxide and silver nanoparticles for synthesis of modified adhesive.

**Figure 2:**
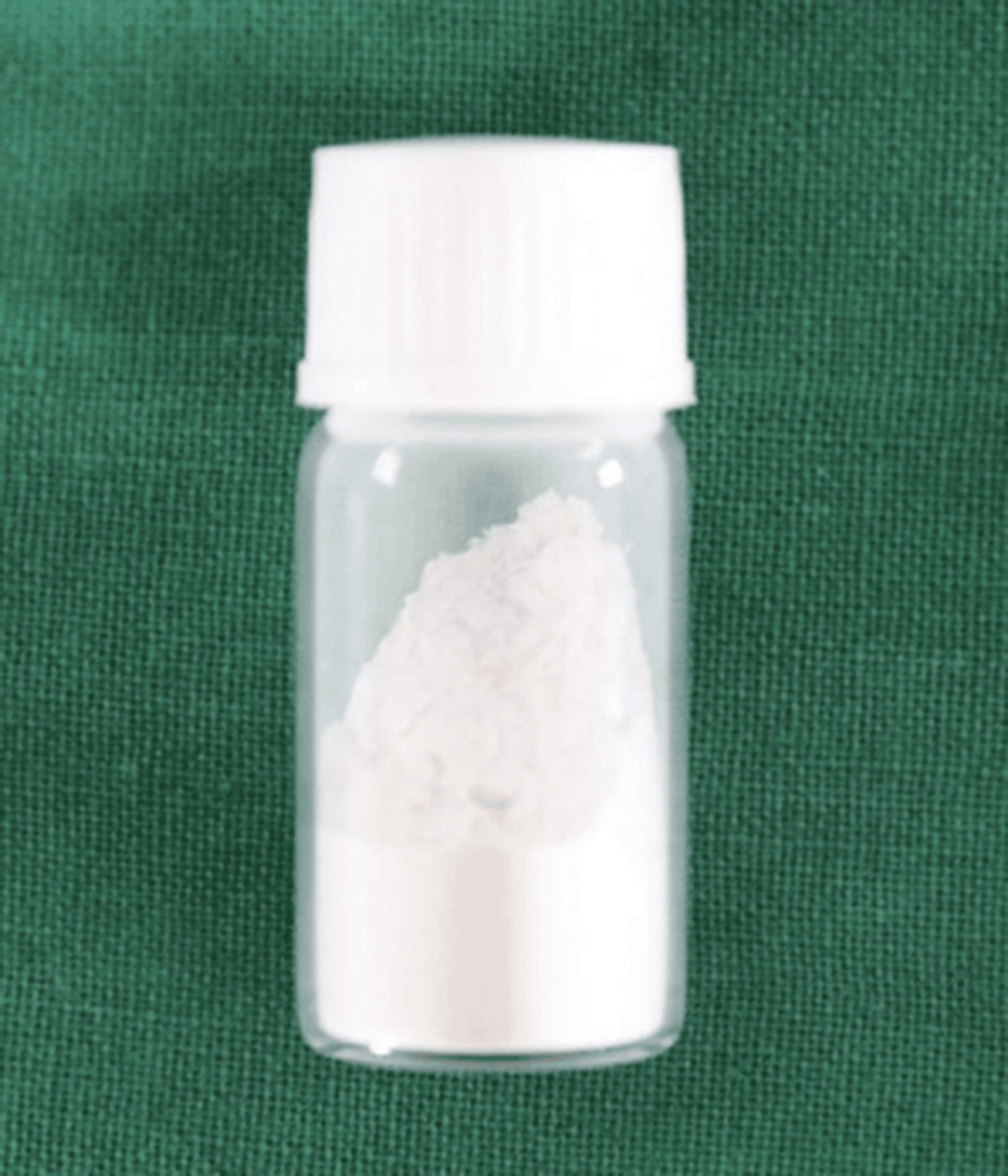
Resveratrol nanoparticles for synthesis of modified adhesive.

Three bottles of commercially available fifth generation, etch and rinse, light cure adhesive primer (3M Unitek Transbond XT; Monrovia, CA: 3M) were incorporated with 1% (w/w) silver nanoparticles (Ag-Np) and zinc oxide nanoparticles (ZnO-Np) (Ultrananotech, Bangalore) (Figure 1). Resveratrol nanoparticles (RSV-Np) were procured from Loba Chemie Pvt. Ltd., India (Figure 2).

The nanoparticles were weighed on an analytical scale and incorporated in three different adhesive solutions in 1% (w/w) and then homogenized by ultra-sonification using ultrasonicator apparatus for 15 min.

Characterization of experimental adhesives 

Following synthesis, the experimental adhesives were characterized at Diya Labs, Mumbai. Scanning electron microscope (SEM) revealed that the nanoparticles were uniformly dispersed in adhesives without agglomeration. Functional group analysis was studied using Fourier transform infrared spectroscopy (FTIR) in the frequency range of 4000-400 cm^-1^.

Sample size

The total sample size of the study was calculated using GPower version 3.1.9.4 (Bonn, Germany: Bonn University). A minimum sample size of 40 was required for shear bond strength testing. The confidence interval was 95%, and the probability of alpha error (level of significance) was 5%. The power of the study was set at 80%.

Statistical analysis

Overall intergroup comparison among four study groups for antimicrobial properties and bond strength was done using the One-way ANOVA F-test followed by Tukey’s post-hoc test for pairwise intergroup comparison between each group. The final sample size consisted of 40 teeth that showed no signs of enamel fracture or bond failure.

Sample collection and inclusion/exclusion criteria

Forty premolar teeth, extracted therapeutically for orthodontic purposes were collected and stored in 0.1% of thymol solution. Healthy and non-hypoplastic extracted teeth free of caries/restoration were included and teeth with attrition, fracture, or iatrogenic damage were excluded. Teeth that underwent bond failure or enamel fracture post-debonding were excluded from the final sample size.

Grouping of sample

The total sample (40) was equally assigned to four groups of n=10 each. There were three experimental groups that were bonded using experimental adhesive and one control group that was bonded using conventional adhesive as described in Table 1.

**Table 1:** Grouping of samples with each group containing 10 premolar teeth.

Groups	Number of premolar teeth (n)	Adhesives
Group 1	10	Silver nanoparticle-reinforced adhesive + conventional composite.
Group 2	10	Zinc oxide nanoparticle-reinforced adhesive + conventional composite
Group 3	10	Resveratrol nanoparticle-reinforced adhesive + conventional composite
Group 4 (control group)	10	Conventional adhesive + conventional composite

Sample preparation for shear bond strength testing

Teeth were mounted vertically in a self-cure acrylic resin block till the cementoenamel junction and crown portion were exposed. The blocks were color-coded in four different colors for easy identification of groups. Each group contained 10 samples that underwent cleaning of the buccal surfaces with a prophylaxis brush. The samples were then washed and tried followed by acid etching using 37% phosphoric acid for 30 seconds, rinsed with running water for 10 seconds, and air dried with air spray for 15 seconds. This was followed by the application of a thin layer of experimental primers in groups 1, 2, and 3, an unmodified primer in group 4, and light cured for 10 seconds each as expanded below.

**Figure 3:**
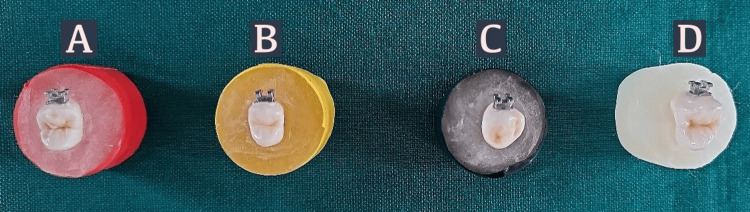
Premolar teeth bonded with MBT brackets embedded in color-coded acrylic blocks for shear bond test. The images show (A) silver NP, (B) zinc oxide NP, (C) resveratrol NP, and (D) control group with unmodified adhesive. NP: nanoparticle; MBT brackets: McLaughlin, Bennett, and Trevisi brackets

Group 1 (Ag-Np; n=10) samples were bonded with metal brackets using silver nanoparticle-reinforced adhesive and conventional composite. Group-2 (ZnO-Np; n=10) samples were bonded with metal brackets using zinc oxide nanoparticle-reinforced adhesive and conventional composite. Group 3 (RSV-Np; n=10) samples were bonded with metal brackets using resveratrol nanoparticle-reinforced adhesive and conventional composite. Group IV (control group; n=10) samples were bonded with metal brackets using conventional adhesive and conventional composite. All the samples were light cured for 30 seconds post-application of 3M Transbond XT light cure composite (Figures 3A-3D).

Assessment of shear bond strength

A blade-end steel rod attached to the machine's crosshead applied an occlusal-gingival load at the enamel-resin interface to test the shear bond strength (SBS). A Universal Testing Machine (model no. UNITEST-10; Pune, India: ACME Engineers) with a crosshead speed of 1.0 mm/min was used to measure bond strength (Pune, India: Praj Laboratories). The value of the maximum load required to debond the bracket was recorded in newtons and converted to megapascals (1 MPa=1 N/mm^2^). The bracket base area was 10.50 mm^2^ (3M Unitek; St. Paul, MN: 3M Orthodontics).

## Results

Shear bond strength analysis

The mean, standard deviation, and 95% confidence interval are represented in Table 2. The highest SBS of orthodontic brackets was present in the control group. Among the experimental groups, Group 3 had the highest SBS, followed by Group 1, with the least in Group 2. The overall intergroup comparison of the three different nanoparticles-incorporated dentin bonding agents on the SBS of orthodontic brackets was calculated using the one-way ANOVA F-test (Table 2). A highly statistically significant (p<0.001) difference was observed among the four groups.

**Table 2:** Descriptive statistics of shear bond strength (MPa) of the four different groups and overall comparison of shear bond strength using one-way ANOVA F-test. *P-value<0.001 was considered a highly statistically significant difference. SE: standard error; MPa: megapascals

Groups	Mean SBS (MPa)	SD	SE	Minimum	Maximum	One-way ANOVA F-test	p-Value
Group 1 (silver nanoparticle)	6.3	0.82	0.26	5.19	7.80	F=28.142	<0.001*
Group 2 (zinc oxide nanoparticles)	5.65	0.83	0.26	3.85	6.85		
Group 3 (resveratrol)	7.6	0.93	0.29	6.21	8.82		
Group 4 (control)	8.6	0.46	0.14	7.92	9.66		

Further, to know the pairwise significant difference between the groups, Tukey’s post-hoc analysis was applied and the results are presented in Table 3. It indicates that group 4 (control group) had highly statistically significantly (p<0.001) greater SBS as compared to groups 1, 2, and 3. Group 3 (resveratrol group) had statistically significantly greater SBS than the other experimental groups.

**Table 3:** Pairwise comparative statistics of shear bond strength (MPa) using Tukey's post-hoc test. *P-value<0.05 was considered significant. **P-value<0.001 was considered highly significant. MPa: megapascals; SBS: shear bond strength

Group	Comparison group	Mean difference between SBS of different groups (MPa)	p-Value
Group 1 (silver nanoparticle)	Group 2 (zinc oxide nanoparticles)	0.65	0.048*
	Group 3 (resveratrol)	1.29	0.004*
	Group 4 (control)	2.30	<0.001**
Group 2 (zinc oxide nanoparticles)	Group 3 (resveratrol)	1.94	<0.001**
	Group 4 (control)	2.95	<0.001**
Group 3 (resveratrol)	Group 4 (control)	1.006	0.034*

## Discussion

Over the years orthodontic adhesives underwent several modifications to their composition and physical properties. Dentin bonding agents (DBA) have seen dramatic improvements in subsequent generations that have improved bond strength to dentin. The fifth-generation adhesives are distinguished as “one-step” or “one-bottle” systems and provide a bond strength of 20 to 30 Mpa. A major disadvantage of acid etching is the demineralization of the enamel surface adjacent to the orthodontic appliance. Along with that, fixed mechanotherapy results in increased areas of food particle retention that leads to a rise in the number of microbial populations [15].

This phenomenon, coupled with the inherent difficulties encountered by patients in maintaining oral hygiene with fixed appliances, contributes to the formation of white spot lesions (WSLs), which persist as a significant clinical concern. These lesions not only compromise the esthetic appearance of patients but also lead to diminished levels of patient satisfaction.

Henceforth, various endeavors have been undertaken to mitigate or regulate the occurrence of white spot lesions. To address these challenges, different modifications have been implemented in orthodontic adhesives, culminating in the development of "experimental composite adhesives" (ECA) [16]. In this new age, there has been a rampant increase in bacterial resistance towards commercially available antimicrobial agents which has in turn led to a crescent need for natural and nontoxic antimicrobial agents [17]. Materials containing fluoride-releasing properties show a rapid decrease in antibacterial activity [18]. Hence, there is a need for novel materials that reduce the adhesion of streptococci to orthodontic adhesives for a prolonged period of time that can in turn prevent enamel demineralization. 

In recent decades, various natural compounds have been studied for their anti-inflammatory and anti-bacterial properties. Among these compounds, trans-resveratrol (RV), chemically denoted as 3,5,4′-trihydroxy-trans-stilbene, a non-flavonoid polyphenol has shown promising results [19]. Studies demonstrate its safety and tolerability, with doses of up to 5 g/day being well-tolerated, whether administered as a single dose or as part of a multiple-day dosing regimen [13,20]. Along with this, metallic nanoparticles are currently considered the gold standard for the most promising agents displaying antibacterial properties that display biocidal activities even at low concentrations [8].

However, the addition of these nanoparticles tends to modify and reduce the mechanical properties of the adhesive. In an attempt to overcome these challenges, this study explored the effect of a natural anti-microbial agent like resveratrol and compared it with the current gold standard, that is, silver and zinc oxide nanoparticles. These nanoparticles were incorporated in dentin bonding agents at a concentration of 1% (w/w) and were tested for their influence on shear bond strength.

The current investigation utilized silver nanoparticles at a concentration of 1.0% in the adhesive formulation due to the documented anti-bacterial properties associated with AgNp when added to dental resins [14]. Furthermore, in alignment with findings by Jatania and Shivalinga, which correlated increased shear bond strength with decreased concentration of zinc oxide (ZnO), a decision was made to incorporate 1.0% ZnO into the adhesive formulation [21].

Our study reported that the addition of Rsv-Np, Ag-Np, and ZnO-Np to dentin bonding agents significantly decreases the mean shear bond strength of DBA. The mean shear bond strength of group 4 (Transbond XT Primer) was significantly higher (p<0.001) as compared to the experimental adhesives. It showed a maximum value of 9.66 Mpa and a minimum of 7.92 Mpa. However, Reynolds has stated that a minimum bond strength of 5.9-7.9 MPa could result in successful clinical bonding [22].

The study recorded group 3 (resveratrol) with the second-highest mean value of shear bond strength, i.e., 7.6 MPa. The minimum recorded value was 6.21 MPa and the maximum was 8.82 MPa. Resveratrol doped adhesive displayed a higher shear bond strength as compared to Ag-Np and ZnO-Np incorporated adhesives. To the best of our knowledge, there is no previous literature that has tested the effect of shear bond strength on resveratrol-doped orthodontic adhesives. The favorable shear bond strength of resveratrol could be due to its collagen crosslinking properties with its phenolic hydroxyl groups. Peng et al. conducted an assessment of the impact of resveratrol solution application as a primer on the coronal dentin to assess resin bond durability. The resveratrol pretreatment group exhibited significantly elevated microtensile bond strength (MTBS), indicative of the superior inhibitory effect on matrix metalloproteinase (MMP) activity [23].

Group 1 (silver nanoparticles) displayed a mean shear bond strength of 6.3 MPa with a minimum value being 5.19 MPa and a maximum is 7.80 MPa. Although Ag-Np adhesive reported a decreased shear bond strength value as compared to the control group and resveratrol Np adhesive, the mean shear bond strength value of Ag-Np fell within the desirable range of 5.9-7.9 MPa. This is in accordance with a study by Reddy et al. where the shear bond strength of orthodontic adhesives was compared between a control group and nanoparticle-incorporated adhesives [14]. It was reported that a significant level of difference between the control group and experimental adhesives was observed with the control group having the highest shear bond strength followed by silver, zinc oxide, and titanium dioxide nanoparticles. Similarly, Degrazia et al. reported a decrease in the shear bond strength of orthodontic adhesive after the incorporation of Ag-Np as compared to the control group [24].

In the present study, Group 2, (ZnO-Np) displayed that the shear bond strength fell within the ideal range but it was comparatively lesser than the control group and the remaining experimental groups of RSV-Np and Ag-Nps. The mean shear bond strength of the dentin bonding agent incorporated with zinc oxide was 5.65 Mpa, the minimum value was 3.85 Mpa and the maximum value was 6.85.

This could be explained by reported previous literature where, Spencer et al. reported that as the concentration of ZnO increases, anti-microbial activity significantly increases [25]. However, they also reported that as the concentration of zinc oxide increases, there is a decrease in shear bond strength. So, the reduced shear bond strength in the present study could be attributed to the concentration of zinc oxide nanoparticles in the experimental adhesives.

Further studies and long-term clinical trials are needed to assess the phenomena of bonding in a better way and discern its effect on human health. Additionally, further research will open new horizons for natural nanoparticles that improve anti-bacterial efficacy without significantly impacting the physical properties of adhesives.

Limitations

The limitations of this study are that it involves an in vitro analysis and further research would require assessing the effect in long-term clinical trials. Animal studies and long-term effect monitoring should be done to improve the utilization of resveratrol in orthodontics.

## Conclusions

Incorporation of nanoparticles into adhesive materials can affect the shear bond strength of adhesives. However, the presented study concluded that resveratrol-incorporated adhesive displayed significantly higher shear bond strength than silver or zinc-incorporated dentin bonding agents. This indicates that resveratrol-doped adhesives can be a better and more natural alternative to synthetic nanoparticles. Further studies can investigate the effect of different concentrations of nanoparticles on other properties.
